# Acceptability, Feasibility, and Appropriateness of the B-OK Bottles as an Implementation Strategy for Treatment Adherence Support by Medical Case Managers

**DOI:** 10.1007/s43477-024-00135-5

**Published:** 2024-09-17

**Authors:** Aaron Richterman, Tamar Klaiman, Rebecca Connelly, Daniel Palma, Eric Ryu, Laura Schmucker, Katherine Villarin, Gabrielle Grosso, Kathleen A. Brady, Harsha Thirumurthy, Alison Buttenheim

**Affiliations:** 1 Division of Infectious Diseases, Department of Medicine, University of Pennsylvania, 3400 Spruce Street, Philadelphia, PA 19104 USA; 2 Leonard Davis Institute of Health Economics, University of Pennsylvania, Philadelphia, PA USA; 3 Department of Medical Ethics and Health Policy, University of Pennsylvania, Philadelphia, PA USA; 4 Division of HIV Health, Philadelphia Department of Public Health, Philadelphia, PA USA; 5 Department of Family and Community Health, University of Pennsylvania, Philadelphia, PA USA

**Keywords:** HIV, Treatment adherence support, Medical case managers, Visual aid, Behavioral economics, Implementation science

## Abstract

Antiretroviral therapy treatment adherence support by medical case managers is an evidence-based practice, but effectiveness may be constrained by limited understanding of antiretroviral therapy’s benefits among people with HIV. We used mixed methods to evaluate the pre-implementation context of the B-OK Bottles (“B-OK”) — a visual aid designed to correct HIV mental models — as an implementation strategy for treatment adherence support by medical case managers in Philadelphia. We assessed outcomes of acceptability, feasibility, and appropriateness among medical case managers and people with HIV. We conducted case manager focus groups at four agencies, and enrolled clients of case managers at these agencies. Clients received the B-OK intervention, a survey, and individual interviews. Among clients, we assessed implementation scales: Acceptability of Intervention Measure, Feasibility of Intervention Measure, and Intervention Appropriateness Measure. During focus groups, medical case managers (*N* = 29) found B-OK to be highly acceptable and feasible, and that it would be appropriate as a conversation starter. Individual interviews (*N* = 52) also demonstrated high degrees of B-OK acceptability, feasibility, and appropriateness for use by case managers. Medical case managers and people with HIV felt that B-OK could improve individual motivation for medication adherence. However, participants also identified other substantial barriers to adherence besides knowledge and understanding. Quantitative results were consistent with our qualitative findings, with high scores on implementation scales. This study suggests that B-OK would be acceptable, feasible, and appropriate as an implementation strategy for treatment adherence support by medical case managers, but that a multifaceted approach is likely needed to achieve optimal adherence.

## Introduction

Non-adherence to antiretroviral therapy and poor retention in care represent the greatest barriers to ending the human immunodeficiency virus (HIV) epidemic in Philadelphia — one of 48 US counties with the highest annual number of new HIV diagnoses that were selected in 2019 as priority jurisdictions by the United States (US) Department of Health and Human Services’ “Ending the HIV Epidemic” (EHE) initiative (Philadelphia Department of Public Health AIDS Activities Coordinating Office, 2020). As of 2020, 25% of people with HIV in Philadelphia who were in care at some point in the previous five years were now out of care, or in care but not virally suppressed (Philadelphia Department of Public Health AIDS Activities Coordinating Office, 2020). These people with HIV are most vulnerable to the adverse health consequences of HIV and, because of the critical importance of HIV treatment as prevention (Cohen et al., 2016; Prevention Access Campaign, 2020; Rodger et al., 2019; Rodger et al., 2016), account for 61% of HIV transmissions in Philadelphia (Philadelphia Department of Public Health AIDS Activities Coordinating Office, 2020). As a result, interventions that can effectively support treatment adherence for this population are crucial to ending HIV epidemics in Philadelphia and elsewhere.

Numerous barriers to antiretroviral therapy have been identified at multiple levels, including individual (e.g., beliefs about antiretroviral therapy), interpersonal/community (e.g., stigma), social/economic (e.g., poverty), and structural (e.g., distance to care facilities) (Ma et al., 2016; Shubber et al., 2016). A variety of supporting interventions have been studied to improve antiretroviral therapy adherence (Kanters et al., 2017; Mbuagbaw et al., 2015; US Centers for Disease Control and Prevention — HIV Prevention Research Synthesis Project, 2022). Among these, evidence-based practices include text messaging reminders, behavioral and cognitive interventions, and supporters (i.e., use of an individual to provide treatment adherence support counseling) (Kanters et al., 2017).

In the US, supporters principally come in the form of medical case managers, who provide a range of individualized services designed to link clients living with HIV with health care, address unmet psychosocial needs, and provide treatment adherence support counseling (HIV/AIDS Bureau & US Department of Health and Human Services, 2013, 2015; Pennsylvania Department of Health, 2021). Medical case managers engage with a diverse array of clients, including those newly diagnosed, lost to care, in care but not virally suppressed, and virally suppressed but struggling with adherence.

Services provided by medical case managers are evidence-based and have proven effective in both establishing and maintaining engagement in care (Craw et al., 2008; Gardner et al., 2005; Harris et al., 2003; Magnus et al., 2001; Rajabiun et al., 2007; Rumptz et al., 2007; Willis et al., 2013). Engagement with medical case managers by people with HIV, including for treatment adherence support, is an evidence-based practice associated with improvements in antiretroviral therapy adherence and engagement and retention in care (Harris et al., 2003; Kushel et al., 2006; Magnus et al., 2001; Rumptz et al., 2007; Willis et al., 2013).

Implementation strategies are needed to maximize the effectiveness of treatment adherence support provided by medical case managers to people with HIV. One clearly identified need is to address the substantial knowledge gaps that persist among people with HIV about the prevention benefits of antiretroviral therapy (Bor et al., 2021; Bor, Onoya, Bor et al., 2021a, b). These remain even in the wake of a global “Undetectable = Untransmittable” (U = U) campaign, which aimed to encourage antiretroviral therapy use through widespread public messaging highlighting that viral suppression with antiretroviral therapy prevents HIV transmission between serodiscordant sexual partners (Prevention Access Campaign, 2020). Randomized controlled trials have shown that interventions that improve understanding of treatment as prevention among people with HIV can improve antiretroviral therapy adherence and viral suppression (Kalichman et al., 2011, 2018).

The B-OK Bottles (hereafter referred to as “B-OK”) are a visual and tactile tool that was developed in South Africa using human-centered design principles and behavioral economics insights to facilitate communication about HIV treatment. B-OK presents health information in a salient manner, leverages framing effects (i.e., that messages about health may be more effective if they do not emphasize illness and vulnerability), and describes HIV pathogenesis using analogies. As a result, B-OK may enable the correction of people’s mental models— simplified cognitive representations of complex concepts that can influence health-related behaviors (Chakrapani et al., 2013; Downs et al., 2008, 2020; Johnson-Laird, 2010; Kealey & Berkman, 2010; Lynam & Brown, 2012; Morgan et al., 2002) — about HIV and antiretroviral therapy, thus enabling people with HIV to better understand the benefits of antiretroviral therapy.

While there is early evidence supporting the acceptability and effectiveness of B-OK in the South African context (Govathson et al., 2023), the appropriateness of B-OK as an implementation strategy for treatment adherence support in high income countries remains unknown. In this study, our objective was to evaluate the pre-implementation context for B-OK in Philadelphia.

## Methods

This study is reported in accordance with The Standards for Reporting Implementation Studies (STaRI) Statement and the Consolidated Criteria for Reporting Qualitative Research (COREQ) (Pinnock et al., 2017; Tong et al., 2007). STaRI includes a 27-item checklist that is aimed to improve reporting of implementation studies. Our focus within this checklist was on the implementation strategy being evaluated (B-OK) (Supplementary Materials).

### Study Overview and Implementation Frameworks

**Fig. 1:**
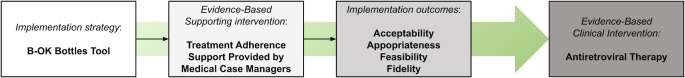
Study overview. Note: Based on the HIV Implementation Outcomes Crosswalk (HIV Implementation Science Coordination Initiative, 2023)

**Fig. 2:**
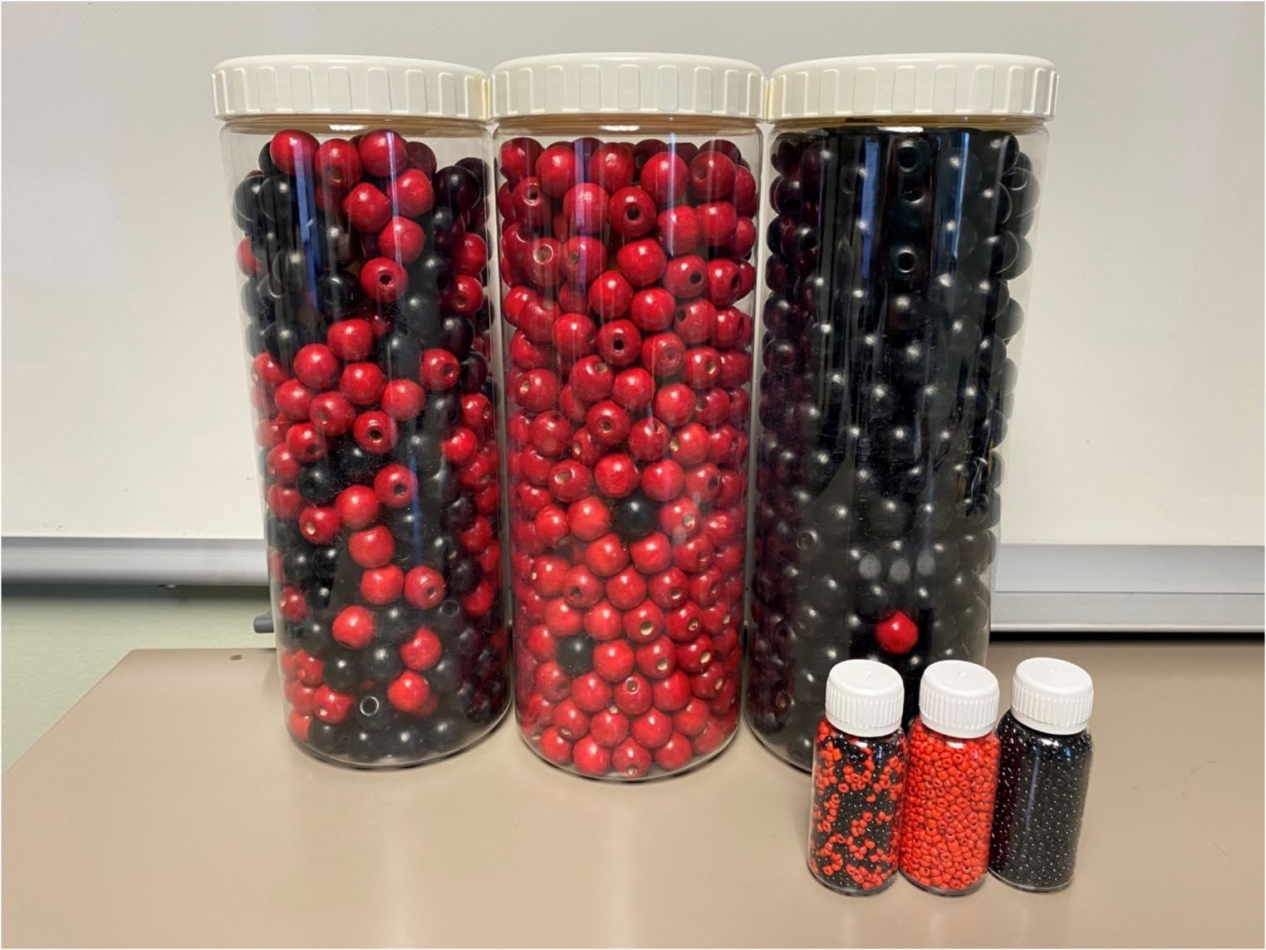
B-OK Bottles. Note: Photo shows two sets of B-OK Bottles — one large, and one small

We conducted pre-implementation contextual inquiry and evaluated the acceptability, feasibility, appropriateness of B-OK as an implementation strategy to improve treatment adherence support among medical case managers (Fig. 1). B-OK is a non-proprietary tool to increase HIV treatment literacy that was developed in South Africa by Population Services International and Matchboxology (Fig. 2) (*Coach Mpilo B-OK Videos*).

The tool includes three bottles containing different combinations of black beads (symbolizing healthy cells) and red beads (symbolizing HIV and HIV-infected cells) that illustrate the human body in the context of HIV infection. One bottle represents the body at the time of diagnosis, with a mix of black and red beads. This visualization communicates that although a person with HIV may be asymptomatic, the red beads signify that HIV is multiplying, detectable, and transmissible. Another bottle contains principally red beads with just a few black beads, serving as an illustration of the potential consequences of delaying or discontinuing treatment. The final bottle, on the other hand, has just one red bead among mostly black beads, demonstrating that daily antiretroviral therapy can almost (but not entirely) eradicate the virus. This bottle shows that the virus is now undetectable and untransmittable — it cannot harm a person with HIV and cannot be transmitted to their partner(s) through sex. We used two sets of bottles — a large set (11 inches tall x 4.5 inches diameter) and a small set (3.75 inches x 1 inch).

B-OK, as a tool designed to facilitate communication about HIV treatment, maps to several implementation strategies identified by the Expert Recommendations for Implementing Change (ERIC) project, including *tailor strategies*, *develop and distribute educational materials*, and *intervene with consumers/patients to enhance uptake and adherence* (Powell et al., 2015). The implementation stages of this project from the HIV Implementation Outcomes Crosswalk are *implementation preparation* and *strategy piloting* (Fig. 1) (HIV Implementation Science Coordination Initiative, 2023). Consequently, we focused on outcomes of acceptability, feasibility, and appropriateness of B-OK as the appropriate outcomes for this study.

The study design was guided by the *implementation planning* approach which combined two implementation science frameworks —The Consolidated Framework for Implementation Research (CFIR) and Reach, Effectiveness, Adoption, Implementation, Maintenance (RE-AIM) — to identify implementation determinants as well as outcomes (Damschroder et al., 2022; Glasgow et al., 1999, 2019; King et al., 2020). These frameworks have been commonly used in other implementation evaluations of interventions to improve antiretroviral therapy adherence (Haberer et al., 2020; Hoskins et al., 2022; Packel et al., 2019). CFIR guided the development of our focus group discussion guides and our interview guides, to ensure that we were addressing the particular domains that are relevant to future B-OK implementation – Innovation, Outer Setting, Inner Setting, and Individuals. Within RE-AIM, we were focused in this study on Reach (understanding of individuals with HIV who are willing to engage with B-OK) and Adoption (understanding of settings and medical case managers who are willing to use B-OK for treatment adherence support). Effectiveness was also addressed but is reported separately (Richterman et al., 2024).

For the purposes of this study, we did not adapt the B-OK Bottles themselves in any way, as part of the objective of this study was to identify areas that required adaptation. We did write a locally appropriate script to be used during the B-OK demonstration.

### Study Design and Population

We conducted a mixed methods study using an intervention (QUAL + quant) design (Creswell & Clark, 2017). We assessed B-OK acceptability, feasibility, and appropriateness among both medical case managers and people with HIV who were clients at four case management agencies in Philadelphia: one community-based organization and three clinic-based organizations.

All medical case managers at participating agencies were invited to participate in focus group discussions. People with HIV who were age 18 years or older and able to speak English or Spanish were referred by medical case managers to study staff who then recruited them for individual study sessions, where they received a B-OK demonstration (5–10 min) and a brief survey questionnaire. They then completed an in-depth qualitative interview and a post-demonstration survey questionnaire that included standard 4-item implementation scales that generate scores from 0 (poorest) to 10 (best): the Acceptability of Intervention Measure, Feasibility of Intervention Measure, and Intervention Appropriateness Measure (HIV Implementation Science Coordination Initiative, 2023; Weiner et al., 2017). Survey items were assessed using probabilistic scales from zero to ten (Preston & Colman, 2000). Study staff observed client interactions with B-OK during the study session as a measure of client engagement with B-OK. We chose to do in-depth interviews (rather than focus groups) for the clients because this would maximize representativeness of a harder-to-reach and often transient population (i.e., no need to return for a scheduled focus group) and minimize confidentiality concerns. We chose to do focus groups for case managers because there was less of a confidentiality concern and this was less of a burden on the agencies while still allowing us to collect high-quality data. We also assessed changes in HIV-related awareness, knowledge, attitudes, intentions, and perceptions among people with HIV after exposure to the B-OK Bottles — these effectiveness outcomes will be presented separately (Richterman et al., 2024).

The University of Pennsylvania Institutional Review Board approved this study. A waiver for consent was granted for medical case manager participants because the only link between the participant and the study would be the consent document and the primary risk was breach of confidentiality. All people with HIV provided written informed consent; one participant withdrew from the study and was not included in this analysis. All participants received $50 compensation.

### Medical Case Manager Focus Group

We conducted one focus group at each of the four case management agencies. E-mail invitations were sent to all medical case managers at each agency, with the timing of the focus group arranged in coordination with agency leadership. Each group was led by a study team member trained in focus group techniques, with a second study team member present to observe and take notes. Focus groups were audio recorded and then transcribed by an external company.

Each focus group began with an introduction to the B-OK Bottles and a description of the project, including a reminder that participation is completely voluntary, and that the discussion would be audio recorded. A focus group discussion guide was used to elicit general reactions to B-OK, thoughts about whether, when, and how to use the bottles to influence patient adherence and other behaviors, perceptions of barriers and facilitators to adherence at multiple levels, and support that would be needed to implement B-OK in medical case managers (see discussion guide in the Supplemental Materials for more details). After each focus group, the study team reviewed the transcript and adjusted the focus group discussion guide to be responsive to the priorities of participants, pursue emerging themes, and probe for additional detail when needed.

### Client Interviews

We recruited people with HIV who were clients of medical case managers consecutively for in-depth interviews focused on implementation outcomes, conducted after a B-OK Bottle demonstration by a study staff. We had a planned sample size of at least 40 interviews, with the final sample size to be determined after saturation of key themes (Vasileiou et al., 2018). We used purposive selection to ensure inclusion across the agencies. We developed a semi-structured interview guide with open-ended questions about the acceptability, feasibility, and appropriateness of B-OK (see interview guide in the Supplemental Materials for more details). One of three study staff members trained in qualitative research (D.P., G.G., and K.V) conducted the interviews in either English or Spanish. Study staff were not employed by the case management agencies and were not involved in the participants’ care. We completed the interviews on the day of enrollment, in person, and in a private space. We conducted interviews until we reached saturation in which three consecutive interviews did not garner new information. Similarly, by the fourth focus group, no additional themes arose which would indicate the need for more data collection. We audio-recorded all interviews, which were then professionally transcribed by an external company (Mcmullin, 2023).

### Qualitative Analyses

The interviewers and focus group moderators were not part of the community from whom data were collected. Results were reviewed by all members of the study team including clinicians and administrative staff to address bias in interpretation of the results.

Thematic analysis was used to analyze focus group data. We used an integrated analysis approach that identified a priori themes summarized across focus groups related to barriers and facilitators to antiretroviral therapy adherence and B-OK (i.e., deductive coding), and also identified themes that arose from the data during at least two focus groups (i.e., inductive coding). Focus group discussion data were organized and analyzed by two investigators (R.C. and E.R.), and illustrative responses were summarized for the team.

Codebooks for the individual interviews with people with HIV were similarly developed using an integrated analysis approach in which themes were identified deductively based on a priori domains related to barriers and facilitators to antiretroviral therapy adherence and B-OK, and inductively based on responses (Chun Tie et al., 2019). Themes were identified when multiple reviewers felt that a concept arose repeatedly. We were specifically interested in concepts that arose across focus groups and individual interviews. We used an iterative process to refine and finalize the codebooks, and individual interviews were independently coded by two investigators (D.P. and E.R.), double-coding 20%. Disagreements were discussed and resolved through consensus. Qualitative analyses were completed using ATLAS.ti version 23 (ATLAS.ti Scientific Software Development GmbH). All representative quotes reflect examples from each theme and were agreed upon by all co-authors.

### Statistical Analyses

We used descriptive statistics to summarize individual item and summary scores for the Acceptability of Intervention Measure, Feasibility of Intervention Measure, and the Intervention Appropriateness Measure. We also used descriptive statistics to summarize participant interactions with B-OK. We performed statistical analysis using SAS Version 9.4 (SAS Institute; Cary, NC).

### Triangulation of Findings

We triangulated data from focus groups and in-depth interviews and found that perspectives among case managers and clients were consistent. As described above, we asked about similar concepts in both interactions. We analyzed each set of data separately, and then compared and reported results by theme to assess variations and overlap (Carter et al., 2014).

In addition, we used methodological integration to assess consistency across methodological approaches (i.e., quantitative component to explain *what* perceptions are, qualitative component to explain *why* these perceptions exist).

## Results

We conducted four medical case manager focus group discussions between 11/2022 and 4/2023 (Agency 1 *N* = 10; Agency 2 *N* = 3; Agency 3 *N* = 10; Agency 4 *N* = 6) for a total of 29 medical case managers. Fifty-two people with HIV completed in-depth interviews. Participants with HIV had a median age of 55 (IQR 50.5–60.5), over half were male sex (58%, *N* = 30), and most spoke English (92%, *N* = 48). Most participants identified as non-Hispanic Black (71%, *N* = 37), with the rest identifying as Hispanic (17%, *N* = 9), non-Hispanic white (6%, *N* = 3), or other or mixed or multiple race/ethnicities (6%, *N* = 3). Nearly half the participants with HIV were clients at the community-based agency (46%, *N* = 24). All participants with HIV were receiving antiretroviral therapy, with all but two prescribed oral antiretroviral therapy (96%, *N* = 50). Among participants receiving oral antiretroviral therapy, the median number of reported doses missed out of the last 30 days was zero (IQR 0–2), and 36 (33%) had sub-optimal adherence (i.e., < 95% self-reported adherence during the last 30 days).

### Barriers and Facilitators to Antiretroviral Therapy Adherence

Respondents from both response groups identified key barriers and facilitators to treatment adherence (Table 1), and medical case managers at all agencies described treatment adherence support as an integral component of their role in caring for people with HIV. Through their experience with treatment adherence support, medical case managers described encountering barriers to adherence at multiple levels among people with HIV. Three out of four agencies brought up health literacy as an important contributor to lack of adherence. According to two out of four agencies, interpersonal stigma also led to nonadherence among people with HIV. In three out of four focus groups, addiction, mental health issues, and history of trauma were identified as important barriers to antiretroviral therapy adherence. Structural and social determinants, like housing, transportation, medication cost, food insecurity, and poverty, were also raised as commonly encountered barriers to antiretroviral therapy adherence in three out of four focus groups.

**Table 1:** Barriers and facilitators to antiretroviral therapy adherence

Theme	Sub Theme	Respondent role	Representative Quote(s)
Barriers	Health Literacy/Education	Participant 30	Oh, my case manager? She don’t talk about this stuff. Because I think some of them need to be educated…The case managers, they don’t talk about it. Because I don’t even think they know about it…They need to be educated, for real.
		Agency 1	With the population that we have, we see a lot of health literacy issues. They might not understand what we’re saying to them, but if they see it, then that will drive the point home
	Stigma	Agency 4	It’s stigma, as well, especially if they haven’t disclosed to their family. They don’t leave their medication out. They have to hide. And life just takes over and they forget
		Participant 25	Some of them, I know they’re probably just scared to ask questions like I was. Or help them, embarrassed, like I should know and they’re gonna judge me
		Participant 76	To be honest with you, I’m real secretive about the virus. Yeah. And for years I was gonna take this to my grave. You have to – I went to the hospital one day and my family went in my room and found my medication. Yeah. After that, I don’t like being around people.
	Mental Health	Agency 4	And then we also have people who struggle with mental health issues, and drug and substance issues. And you come – they come in and they get an emergency supply of meds, and then they call you five hours later and say, oh, I left it on the bus.
		Participant 71	And just by watching the public out there just, I mean, I see young people, younger than you, shooting needles up and they don’t care where they get them from. And then you see them nodded down, the next thing you know they’re either dead or gonna die from the virus that they – because they don’t know what they’re putting in their system.
	Social and Structural Determinants	Agency 1	Sometimes it’s transportation. We find that it can be a barrier, so we try to provide bus passes or, depending on the insurance, rides to and from medical appointments
		Agency 2	It doesn’t matter if it’s a dollar or three dollars, most of our patients do not have the copay. […] If they only had one medication, maybe the pharmacy may be like, okay, I’ll waive the fee. But if they have the HIV med and plus ten other medications, that might not happen
	Medication Side Effects	Participant 17	Well, there used to be a time where people never took them, because of the side effects of them. You would feel blue or just psychotropic – traces of like depression and some people would commit suicide and they – back in the ‘90s, that was the situation with me, but I always go back to talking to my doctor. I didn’t like them.
Facilitators	U = U	Agency 3	At least for me in the therapy world, I find that U = U is something that allows clients to have hope that they can still have a full and fulfilling life outside of the diagnosis. Because I think like [one of the other medical case managers] shared, most people when you get this diagnosis, you’re combated with stigma, fear, all the things. And knowing that if you take your medicine, you get to undetectable status, you’re still able to have a happy, healthy sex life as the next person. So that gives people some level of hope.
		Participant 5	Well, your viral load is undetectable so you can’t really pass it to that person. It’s because if you’re taking your medicine like you’re supposed to, you’re not passing it on to that person. But, if you don’t take your medicine, you can pass it.
	Education	Participant 50	And it’s a lot of kinda self-education on it, or whatever. It’s not a lot of classes or – I mean, yes, other than going to the doctors and they tell you information. But I think more classes or more education one on one, or something like that, you can retain the information better to be knowledgeable about it.

Note. Table shows emergent themes and representative quotes from medical case manager focus groups (*N* = 4 focus groups with a total of 29 medical case managers) and individual in-depth interviews with clients with HIV (*N* = 52)

Participants with HIV also highlighted barriers to antiretroviral therapy adherence. Some felt internalized stigma associated with HIV and felt that discomfort sharing their status could affect their adherence. Side effects of antiretroviral therapy medication had historically impacted some participants’ adherence as well, although this predominantly applied to older antiretroviral regimens with lower tolerability. Participants felt that, at times, interpersonal stigma impacted their adherence to antiretroviral therapy. Social and structural barriers to adherence included substance use and unstable housing; however, case managers helped to mitigate some of the social barriers their clients experienced.

Two out of four focus groups suggested that most people with HIV understand elements of the concept of U = U, and two out of four focus groups indicated that medical case managers have found U = U to be a helpful motivator for antiretroviral therapy adherence. Clients with HIV who reported understanding U = U prioritized adherence to stay undetectable and prevent transmission.

Some participants with HIV felt that education would be helpful in ensuring patients understand their diagnosis and treatment options, and optimizing adherence. Participants with HIV reported heterogeneous experiences with prior treatment adherence support. Some also mentioned the benefits of support groups in helping them cope with their status and reinforced the need to take medication daily.

### Acceptability, Feasibility, and Appropriateness of B-OK Bottles

All focus groups found B-OK to be highly acceptable and feasible for use by medical case managers, and reported that visual tools were currently unavailable and needed to facilitate treatment adherence support (Table 2). Three out of four focus groups expressed that B-OK (in particular the larger bottles) would be attention-grabbing to people with HIV.

**Table 2:** B-OK bottle acceptability, feasibility, and appropriateness

Theme	Sub-Theme	Respondent role	Representative Quote(s)
Acceptability	Appearance	Participant 19	The display, basically, it’s really simple. I mean, you got the mixed beads in there and that’s telling me that you just got virus, the red is saying that you got AIDS or close to it and the black’s saying you in – you’re undetectable, basically.
		Agency 4	I really like it. The first reaction I had was wow, that’s a big container. Then you pulled out that, and then it was helpful because if I was in a group, and I pulled that out, I would have attention. Everyone would be like, what are you doing? What is that?
		Agency 3	I was going to say, if I was [living with HIV], just by looking at those bottles, I kind of don’t need to understand what viral load means. I kind of really don’t need to understand what CD4 means. If you tell me, this is where I’m at right now and if you take your medication without worrying about all the correct terms or anything like that, because that’s where people get lost at. I feel just by this visual itself, even just makes me personally feel like I need to take my medications because I want to get to this bottle [pointing at bottle with all black beads with one red bead].
Feasibility	Likelihood of use	Agency 3	If someone said, would you like these? I’d say, yep. I’d put it in my bag. And then if I was ever having – if I ever was in the middle of a conversation and I was like, we are having a barrier here. I’m not sure if you’re asking – I’d literally just pull them out and say, let’s just go over your viral load and information
		Participant 5	So I think every hospital and every doctor, every case manager, they should have this in their rooms and just explain it to us. That’s what I think.
Appropriateness (Populations)	Conversation Starter	Agency 2	So, for patients who are very familiar, very comfortable with lingo and everything else, I feel like it would be more of a conversation piece as far as being like, hey, look at these.
	Not virally suppressed	Agency 1	The folks who aren’t virally suppressed … are the people the managers will want to actually have this visual with them and saying, hey, guys, this is where you are at and my goal is – first off, figure out what the barrier is that they’re not taking the meds. Right? And once you guys figure that out, then you can kind of show them a visual.
	Newly Diagnosed	Agency 2	I think with newly diagnosed patients, it’s a lot [of] information up front. And I think a lot of it, it gets lost during the process, because they’re having to first deal with the diagnosis, understand how to navigate through life after that. And with the U = U, I’ve noticed some patients don’t believe it, because they haven’t encountered HIV before so they’re not up on treatment or know the new advances. So I think seeing the visual will definitely help them as they’re navigating and learning to understand how HIV is actually affecting their body
	Support Groups	Agency 1	And even in our support groups. Because we have coed and women’s support groups, right, so we can even show them that – and especially our women’s support group, they’re very active, so kind of show – go over it again, offer a refresher.
	English as a second language	Agency 4	I immediately thought of a conversation I had with a patient where we’re having communication issues, even with an interpreter […] and she was not understanding the information […] when talking with a patient who has a lower level of understanding as far as the condition and everything else, especially when you have multiple things being explained in the same visit, it would be helpful to have a prompt to be like, okay. Now we’re talking about HIV.

Note. Table shows emergent themes and representative quotes from medical case manager focus groups (*N* = 4 focus groups with a total of 29 medical case managers) and individual in-depth interviews with clients with HIV (*N* = 52)

Medical case managers from all focus groups thought that B-OK could serve medical case managers well as a conversation starter about antiretroviral therapy adherence. In terms of the appropriate population of people with HIV for B-OK, two focus groups identified people with HIV who were not virally suppressed as likely to benefit. Two out of four focus groups also pointed to people with HIV who were newly diagnosed as an important context to consider the use of B-OK. Three out of four agencies indicated that B-OK would be useful for people with HIV with English as a second language, or with low health literacy. In addition to individual counseling sessions, three out of four focus groups indicated that B-OK would be appropriate during larger support group discussions held at the agencies. Participants with HIV liked the appearance of the bottles and thought the red and black colors quickly clarified the impact of the virus on the body and medicine on the virus. Many participants with HIV felt that case managers should have B-OK to show their patients, and that B-OK would be highly appropriate for that context. Some participants with HIV found B-OK so helpful that they wanted their own set to share with peers.

Medical case managers in all focus groups suggested minor changes to improve B-OK, such as sealing the bottles so they didn’t spill, creating additional sizes, changing the colors (although the current colors were preferred by most participants), adding an accompanying pamphlet or digital resource, or providing an accompanying script for medical case managers to use during counseling. B-OK acceptability was further illustrated by medical case managers from all agencies immediately requesting a set of bottles, asking if they could take photos of the bottles to share or to use to make their own set of bottles.

### Quantitative Data among People with HIV

Quantitative results were consistent with our qualitative findings, with clients reporting extremely high degrees of B-OK acceptability on the Acceptability of Intervention Measure (median score 10 out of 10, IQR 9.75 to 10), feasibility on the Feasibility of Intervention Measure (median score 10 out of 10, IQR 10 to 10), and appropriateness on the Implementation Appropriateness Measure (median score 10 out of 10, IQR 10 to 10). Engagement with B-OK during the study session was also high based on participant interactions with the bottles (Table 3).

**Table 3:** Engagement with B-OK bottles

	Number of participants	Percent of participants
When bottles are first put in front of participant		
The participant looks at the bottles	52	100%
The participant touches/picks up the bottles	8	15%
The participant asks questions about the bottles	5	10%
The participant requests a set of bottles	1	2%
The participant refers to the bottles during discussion	4	8%
**During the B-OK Bottles demonstration**		
The participant looks at the bottles	52	100%
The participant touches/picks up the bottles	27	52%
The participant asks questions about the bottles	14	27%
The participant requests a set of bottles	1	2%
The participant refers to the bottles during discussion	18	35%
**After the B-OK Bottles demonstration**		
The participant looks at the bottles	51	98%
The participant touches/picks up the bottles	22	42%
The participant asks questions about the bottles	12	23%
The participant requests a set of bottles	1	2%
The participant refers to the bottles during discussion	28	54%
**Any time**		
The participant looks at the bottles	52	100%
The participant touches/picks up the bottles	34	65%
The participant asks questions about the bottles	24	46%
The participant requests a set of bottles	3	6%
The participant refers to the bottles during discussion	35	67%

Note. Total sample size is 52 clients with HIV

## Discussion

In this mixed methods study of medical case managers and their clients with HIV, we found that B-OK would be highly acceptable, feasible, and appropriate for use as an implementation strategy for the evidence-based practice of treatment adherence support by medical case managers, and that preliminary findings of B-OK engagement were high among people with HIV. These findings support B-OK being translatable to a US urban setting, outside of the South African context where it was originally developed and tested (Govathson et al., 2023). Medical case managers in this study did not report using other specific interventions or tools during treatment adherence support. Our study population reflected the demographics and racial composition of people with HIV in Philadelphia (Philadelphia Department of Public Health AIDS Activities Coordinating Office, 2021). While we did not collect detailed socioeconomic data, all participants were seen at Ryan White-funded case management sites and therefore met Ryan White eligibility by having income less than 500% of the federal poverty level.

B-OK has several strengths as an implementation strategy for treatment adherence support. It is a visual tool can be used with patients with low literacy and numeracy, and can facilitate the use of metaphor, analogy, and narrative (Arroliga et al., 2002; Casarett et al., 2010). Incorporating B-OK into treatment adherence support can help correct faulty or outdated mental models of HIV treatment effectiveness and the importance of adherence; can reframe and re-focus attention on the benefits of achieving viral suppression and away from the loss of health; and can improve retention, recall, and comprehension of complex clinical topics. B-OK is low-cost, easy to construct, and does not require technical terminology for use. Consequently, we hypothesize that B-OK will be easily scalable with high potential for sustainment over time, although these outcomes were not measured in the current study. Visual tools have shown promise for improving HIV adherence in other clinical contexts (Kalichman et al., 2013), although evidence remains limited. This is the first study of which we are aware to examine implementation aspects of such a tool.

Findings from this pre-implementation contextual inquiry can be mapped onto the Innovation, Inner Setting, Individuals, and Outer Setting CFIR domains to inform future B-OK implementation (Damschroder et al., 2022). In each of these domains, our findings support the essential components of B-OK as an implementation strategy, and show how B-OK has strong potential to facilitate treatment adherence support. As this was a study of the pre-implementation context, we did not evaluate the Implementation Context CFIR domain (this will be addressed in future work).

In the Innovation domain (i.e., B-OK itself), our study found that no similar visual tools are currently available for use by medical case managers during treatment adherence support. Both medical case managers and people with HIV found B-OK to be highly appealing for use in this context. Furthermore, B-OK was deemed adaptable to meet the specific needs of people with HIV, aligning well with the multifaceted role of medical case managers. Participants also considered B-OK to be straightforward and intuitive as a tool to explain complex biological concepts related to HIV. Though not directly assessed during this study, B-OK is easy to construct, and the cost of production is minimal. To address questions of B-OK effectiveness, we also assessed changes in HIV-related awareness, knowledge, attitudes, intentions, and perceptions among people with HIV after exposure to B-OK— these findings will be presented separately.

There were several relevant findings that mapped to the Inner Setting domain — within the agency itself. Treatment adherence support was described as a major part of medical case managers’ work, although people with HIV noted that treatment adherence support was frequently sub-optimal. Medical case managers felt that B-OK would fit well into their workflows, both in individual and group counseling sessions, since it would take only a short amount of time for medical case managers to explain B-OK to their clients. The relative priority of B-OK and the readiness for implementation were highlighted by immediate requests for the bottles by medical case managers (and some people with HIV).

The Individuals domain refers to findings related to the medical case managers (B-OK deliverers) and people with HIV (B-OK recipients) themselves. Medical case managers were found to have the opportunity and motivation to provide effective treatment adherence support, though some people with HIV felt that additional education about HIV and U = U would improve their capability. People with HIV noted that, in some cases, medical case managers did not fully understand adherence barriers they faced. In these cases, B-OK may help facilitate these discussions. Notably, the concept of treatment as prevention (U = U) was described as a potential motivator for adherence by both medical case managers and people with HIV. Importantly, however, other substantial individual barriers to adherence were noted by both medical case managers and people with HIV, including internalized stigma, substance use, and mental health issues.

The Outer Setting domain in this study refers to everything occurring outside of the medical case management agency, primarily encompassing various barriers to adherence. Notable barriers included interpersonal stigma, and structural and social determinants of health (housing, transportation, medication cost, food insecurity, poverty). The existence of these substantial barriers to adherence at multiple levels suggests that B-OK (or treatment adherence support) alone is unlikely to lead to major improvements in adherence and should be considered as part of combination and personalized approaches that also address these barriers.

In addition, in contrast to the one-time B-OK sessions used for the purposes of the study, B-OK is likely to be most helpful when used in a longitudinal manner that reinforces HIV mental models related to the bottles. This kind of longitudinal strategy will involve an initial in-depth medical case manager educational session (similar to this study, except customized to the patient circumstance — new diagnosis, struggling with adherence, etc.), visual reminders or cues provided to the patient (e.g., a B-OK keychain), and follow-up “check in” sessions by medical case managers to see where a patient envisions themselves currently along the B-OK bottle spectrum.

## Limitations

This study has several limitations. Although one-third of participants reported sub-optimal adherence, self-reported rates of antiretroviral therapy adherence were generally high. This is notable in the context of an implementation strategy that is most likely to be of greatest use among people struggling with adherence. As B-OK is implemented in the future, it will be important to further consider perspectives of people with HIV with lower rates of adherence.

This study was conducted in a single US urban center, although we recruited participants from agencies with diverse practice contexts within the US. Our study was designed to assess the pre-implementation context, and therefore longitudinal and long-term implementation outcomes remain unknown. Our study did not include an intersectional analysis, which incorporates how intersecting social and structural factors and systems of power can influence implementation (e.g., how structural racism impacts how different racialized groups experience treatment adherence support counseling) — this can be considered in future work (Presseau et al., 2022).

## Conclusion

Taken together, our findings provide important pre-implementation context for future B-OK implementation and suggest that B-OK would be highly acceptable, feasible, and appropriate for use as an implementation strategy for treatment adherence support by medical case managers. While B-OK could improve individual motivation for antiretroviral therapy adherence by improving understanding of HIV treatment as prevention and correcting mental models about HIV more generally, the existence of other substantial barriers to adherence at multiple levels suggests the necessity for a combination and personalized approach to achieving optimal antiretroviral therapy adherence. Within the RE-AIM framework, this pre-implementation study addresses likelihood of future Reach and Adoption, with Effectiveness reported separately (Richterman et al., 2024), implying that next steps should include additional evaluations of Effectiveness (focused on clinical outcomes) as well as Implementation and Maintenance.

## Data Availability

Data are available upon reasonable request to the senior author.

## Abbreviations

AIM: Acceptability of Intervention Measure
B-OK: B-OK Bottles
CFIR: Consolidated Framework for Implementation Research
EHE: Ending the HIV Epidemic
EPPM: Extended Process Parallel Model
ERIC: Expert Recommendation for Implementing Change
FIM: Feasibility of Intervention Measure
HIV: human immunodeficiency virus
IAM: Intervention Appropriateness Measure
RE-AIM: Reach Effectiveness Adoption Implementation Maintenance
STaRI: Standards for Reporting Implementation Studies
US: United States
U = U: undetectable = untransmittable
COREQ: Consolidated Criteria for Reporting Qualitative Research
